# Deep learning-based electroencephalic diagnosis of tinnitus symptom

**DOI:** 10.3389/fnhum.2023.1126938

**Published:** 2023-04-26

**Authors:** Eul-Seok Hong, Hyun-Seok Kim, Sung Kwang Hong, Dimitrios Pantazis, Byoung-Kyong Min

**Affiliations:** ^1^Department of Brain and Cognitive Engineering, Korea University, Seoul, Republic of Korea; ^2^Biomedical Engineering Research Center, Asan Medical Center, Seoul, Republic of Korea; ^3^Department of Otolaryngology, Hallym University College of Medicine, Anyang, Republic of Korea; ^4^McGovern Institute for Brain Research, Massachusetts Institute of Technology, Cambridge, MA, United States; ^5^Department of Brain and Cognitive Sciences, Massachusetts Institute of Technology, Cambridge, MA, United States; ^6^Institute of Brain and Cognitive Engineering, Korea University, Seoul, Republic of Korea

**Keywords:** tinnitus, electroencephalography, diagnosis, classification, deep learning

## Abstract

Tinnitus is a neuropathological phenomenon caused by the recognition of external sound that does not actually exist. Existing diagnostic methods for tinnitus are rather subjective and complicated medical examination procedures. The present study aimed to diagnose tinnitus using deep learning analysis of electroencephalographic (EEG) signals while patients performed auditory cognitive tasks. We found that, during an active oddball task, patients with tinnitus could be identified with an area under the curve of 0.886 through a deep learning model (EEGNet) using EEG signals. Furthermore, using broadband (0.5 to 50 Hz) EEG signals, an analysis of the EEGNet convolutional kernel feature maps revealed that alpha activity might play a crucial role in identifying patients with tinnitus. A subsequent time-frequency analysis of the EEG signals indicated that the tinnitus group had significantly reduced pre-stimulus alpha activity compared with the healthy group. These differences were observed in both the active and passive oddball tasks. Only the target stimuli during the active oddball task yielded significantly higher evoked theta activity in the healthy group compared with the tinnitus group. Our findings suggest that task-relevant EEG features can be considered as a neural signature of tinnitus symptoms and support the feasibility of EEG-based deep-learning approach for the diagnosis of tinnitus.

## 1. Introduction

Tinnitus is the illusory perception of sound in the absence of an external sound (Jastreboff and Sasaki, 1994; Baguley et al., 2013; Mohamad et al., 2016). People with tinnitus experience impaired cognitive efficiency and difficulties in mental concentration (Hallam et al., 2004). Based on functional imaging studies, it is generally accepted that tinnitus is associated with maladaptive neuroplasticity because of impairment in the auditory system (Schaette and McAlpine, 2011; Faber et al., 2012; Roberts et al., 2013; Kaya and Elhilali, 2014; Hong et al., 2016; Ahn et al., 2017). Most symptoms of tinnitus can be attributed to reorganization and hyperactivity in the auditory central nervous system (Muhlnickel et al., 1998; Kaltenbach and Afman, 2000; Salvi et al., 2000; Eggermont and Roberts, 2004). Tinnitus perception can be subject to top-down modulation of auditory processing (Mitchell et al., 2005) or attentional bottom-up processes that are influenced by stimulus salience. We reported neurophysiological and neurodynamic evidence revealing a differential engagement of top-down impairment along with deficits in bottom-up processing in patients with tinnitus (Hong et al., 2016). In addition, we observed that fronto-central cross-frequency coupling was absent during the resting state (Ahn et al., 2017), reflecting that maladaptive neuroplasticity or abnormal reorganization occurs in the auditory default mode network of patients with tinnitus.

Due to many possible causes, such as abnormality in top-down or bottom-up processes, and different symptoms, such as hearing loss or noise trauma, there is currently no universally effective clinical method for tinnitus diagnosis (Hall et al., 2016; Liu et al., 2022). At present, the diagnosis battery for tinnitus relies mainly on subjective assessments and self-reports, such as case history, audiometric tests, detailed tinnitus inquiry, tinnitus matching, and neuropsychological assessment (Basile et al., 2013; Tang et al., 2019).

Neuroimaging techniques are widely applied to monitor neural activity and diagnose different brain disorders. Electroencephalography (EEG) has emerged as one of the most practical techniques since it gives an insight into the temporal neuro-dynamics, it has an excellent temporal resolution (milliseconds or better), good portability, and an inexpensive set-up cost in comparison to other neuroimaging techniques, such as magnetoencephalography (MEG) or functional magnetic resonance imaging (fMRI) (Min et al., 2010). Many researchers have proposed that the assessment of abnormal neural activity as assessed by EEG signals may aid in the diagnosis of tinnitus since this disease is often associated with changes in the brain. Specifically, it is hypothesized that subjective tinnitus is the result of abnormal neural synchrony and spontaneous firing rates in the auditory system, therefore an EEG-based diagnostic approach for tinnitus may be an objective method to evaluate or predict its symptoms (Ibarra-Zarate and Alonso-Valerdi, 2020).

Here, to investigate whether patients with tinnitus can be identified using top-down or bottom-up EEG features, we used an active oddball paradigm (as a top-down directed task) in comparison to a passive oddball paradigm (as a bottom-up directed task). Task-relevant modulations may be reflected in event-related oscillations and provide the essential electrophysiological features for identifying patients with tinnitus. Thus, in the present study, we extracted top-down and bottom-up EEG cognitive signals and used them as discriminative features to identify patients with tinnitus using a novel deep-learning-based tinnitus-diagnostic tool.

Recent studies used machine learning to reduce reliance on experts and mitigate the influence of personal factors in the tinnitus-diagnosis process (Li P.-Z. et al., 2016; Wang et al., 2017; Sun et al., 2019). However, most machine learning EEG studies in tinnitus used resting-state EEG and focused on model performance or methodology (Mohagheghian et al., 2019; Sun et al., 2019; Allgaier et al., 2021). Therefore, there is a need for additional research on the diagnostic efficacy of EEG not only in resting-state but also in task-based studies (Mohagheghian et al., 2019; Allgaier et al., 2021). We hypothesized that there would be differences in brain activity during auditory cognitive tasks, reflecting distinct brain processing mechanisms between healthy individuals and patients with tinnitus. To assess, we used time-frequency analysis of EEG signals during task performance and evaluated neurophysiological differences in EEG spectral activity between the healthy and tinnitus groups. Importantly, we discriminated patients with tinnitus from healthy individuals using a deep learning decoding model and investigated whether the features learned by the model were consistent with task-relevant neurophysiological correlates.

## 2. Materials and methods

### 2.1. Participants

Eleven patients with tinnitus (six women; mean age 32.1 years) and 11 age-matched healthy volunteers (five women; mean age 27.2 years) participated in the experiment. All patients had definite signs of chronic tinnitus, which lasted longer than 3 months but less than a year but had normal hearing otherwise. We assessed normal hearing with the following criteria: (i) the audiometric threshold was within 25 dB of the pure tone average at octave frequencies within 250–8,000 Hz; (ii) transient-evoked otoacoustic emissions (TEOAEs) were recorded in the external ear canal after stimulation with at least 5 dB signal-to-noise ratio (SNR), and distortion product otoacoustic emissions (DPOAEs) were recorded with at least 3 dB SNR; (iii) peak latencies for waves I-III were less than 2.4 ms, and for wave V were less than 6.2 ms on 90 dB normalized hearing level click-evoked auditory brainstem responses (ABR); and (iv) the tympanic membrane had a normal appearance on otoscope examination. Note, normal wave I-III latencies typically suggest intact peripheral auditory nerves (Moller et al., 1981; Moller and Jannetta, 1982), and normal otoacoustic emissions typically suggest normally functioning cochlear hair cells (Kemp, 1978; Mills and Rubel, 1994). However, it is possible that deafferentation was present in some of the tinnitus patients, though not detectible by conventional tests (TEOAEs, DPOAEs, ABR, and otoscope examination).

In addition, we introduced the following exclusion criteria to match patients and healthy volunteers in cognitive abilities: (i) age older than 50 years; (ii) present or past diagnosis of vertigo, Meniere’s disease, noise exposure, hyperacusis, or psychiatric problems; (iii) exposure to ototoxic drugs; (iv) complex cases of tinnitus, for example, a failure of tinnitus pitch matching.

To assess tinnitus severity, all patients completed a tinnitus questionnaire with a 0–10 scale (0: no annoyance; 10: severe annoyance) and a Korean translation of the Tinnitus Handicap Inventory of the American Tinnitus Association (Newman et al., 1996). The healthy volunteers had normal hearing and no signs of tinnitus.

Note, the data in this study were previously collected and published by our group. Extended details on the participants, data acquisition, and audiometric and tinnitus tests are provided in our previous study (Hong et al., 2016).

### 2.2. Materials and procedure

**FIGURE 1:**
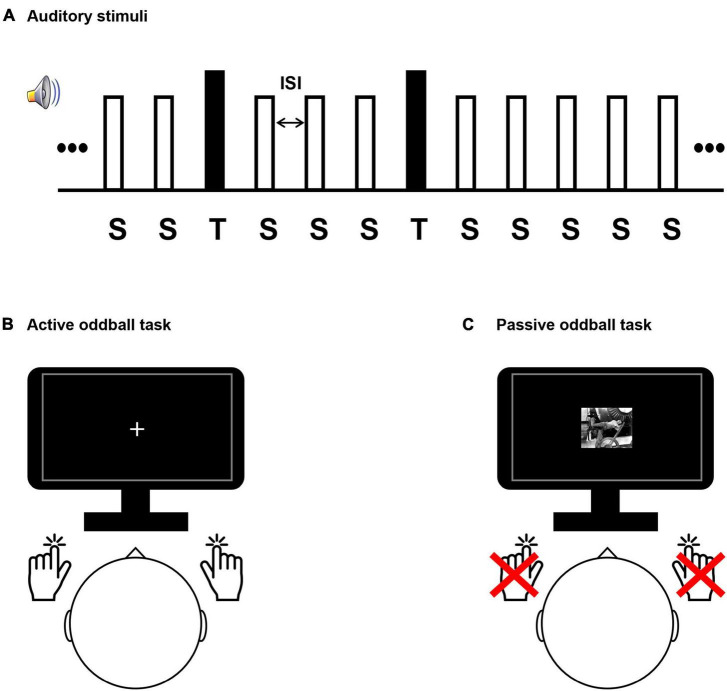
Experimental design. **(A)** The auditory stimulus presentation sequence comprised a series of frequent standard “S” stimuli (80% occurrence probability; 500 Hz tones) and rare target “T” stimuli (20% occurrence probability; 8 kHz tones for healthy subjects and individual tinnitus pitch-matched frequencies for patients). Stimuli were presented for 200 ms and had variable interstimulus interval (1,300–1,700 ms). Participant conducted a sound discrimination task in the active oddball task **(B)**, and passively watched a silent movie in the passive oddball task **(C)**.

Participants performed auditory active and passive oddball tasks during EEG acquisition (Figure 1). During the active oddball task, two auditory stimuli (standard and target stimuli) were presented in random order for 200 ms, with standard stimuli being more frequent than target stimuli by an 8:2 ratio. Participants were instructed to discriminate between the frequently occurring standard stimuli and infrequently occurring target stimuli by pressing a button. We employed an active oddball task because the P300 component of the event-related potential (ERP) reflects fundamental cognitive processes (Donchin and Coles, 1988; Johnson, 1988; Picton, 1992; Polich, 1993, 2007) and is strongly elicited by this task (Katayama and Polich, 1999). Specifically, the P300 component is known to be involved in the contextual updating process (Polich, 2003). In the active oddball task, the P300 elicited by the target stimulus is a large, positive potential that is strongest over the parietal electrodes and occurs at about 300 ms post-stimulus in healthy young adults. Because the active oddball task required the participants’ active responses and therefore engaged cognitive decision-making processes, the results were interpreted as mostly auditory top-down effects. On the other hand, the same stream of auditory stimuli used in the active oddball task was also passively heard by the participants and was principally interpreted as a bottom-up process. This is because auditory bottom-up attention is a sensory-driven selection mechanism for shifting perception toward a salient auditory subset within an auditory scene (Kaya and Elhilali, 2014). In the passive oddball task, a mismatch negativity (MMN) component of ERP would be elicited. Since it is observed even if subjects do not perform a task using the stimulus stream, the MMN is a relatively *preattentive* and *automatic* response to an auditory stimulus deviating from the preceding standard stimuli (Näätänen and Kreegipuu, 2012). As deviant stimuli (physically the same as the target stimuli in the active oddball task) would evoke greater negative potentials compared to standard stimuli, with a fronto-central scalp maximum around 200 ms post-stimulus, this discrepancy is often isolated from the rest of the ERPs with a deviant-minus-standard difference wave, which is called an MMN.

Participants performing the active oddball task were required to respond by pressing a button with one hand when a standard stimulus was detected and another button with the opposite hand when an infrequent target stimulus was detected (Duncan-Johnson and Donchin, 1977; Polich, 1989; Verleger and Berg, 1991; Figure 1). While the participants performed the active oddball task, they were instructed to fixate their eyes on a cross presented at the center of a screen to minimize any possible distracting effects due to alterations in visual attention. The passive oddball task presented the same auditory stimuli as the active oddball task, but participants had to remain still without pressing the button. In addition, to distract from attending to the auditory stimuli, participants performing the passive oddball task were shown a black-and-white silent movie (“Modern Times”: Charlie Chaplin’s movie).

The distance between the participant and the monitor was 80 cm, and visual stimuli were displayed within a visual angle of 6.5° to avoid image formation in the blind spot. The frequencies of the target stimuli in the oddball paradigm for the tinnitus group were their individual tinnitus-pitch-matched frequencies, and the target-stimulus frequency of the healthy group was 8 kHz, which was the most prominent as the individual tinnitus frequency in the tinnitus group. It has been reported that tinnitus patients respond sensitively to tinnitus sound stimuli (Cuny et al., 2004; Li Z. et al., 2016; Milner et al., 2020). The standard-stimulus frequency for both groups was 500 Hz. The experimental paradigm consisted of 320 standard stimuli (80% occurrence probability in the stimulus set) and 80 target stimuli (20% occurrence probability in the stimulus set), which were presented in random order. To minimize temporal expectancies, the interstimulus intervals (ISIs) were set to have variable intervals, ranging randomly between 1,300 and 1,700 ms, and centered at 1,500 ms (Min et al., 2008). All auditory stimuli were generated through Adobe Audition software (version 3.0, Adobe Systems Incorporated, San Jose, CA), and were presented through insert-earphones (EARTONE 3A^®^, 3M Company, Indianapolis, IN, USA) in both ears of the participants. All participants performed the task in the same environment, and the acoustic intensities of all stimuli were set to 50 dB SPL (sound pressure level) using a sound level meter (Type 2250, Brüel and Kjör Sound and Vibration Measurement, Denmark).

### 2.3. EEG recordings

EEG signals were recorded using a BrainAmp DC amplifier (Brain Products, Germany) with a 32 Ag/AgCl-electrode actiCAP having a 10–10 electrode system placement. The ground was set as the AFz electrode, and the reference was set as an electrode on the tip of the nose. The impedances of the electrodes were lowered below 5 kΩ during electrode setup. EEG data were collected with a sampling frequency of 1 kHz and an analog band-pass filter of 0.5–70 Hz. An electrooculography (EOG) electrode was placed below the left eye to track eye movement artifacts. Vertical and horizontal electroocular signals were then estimated using the Fp1–EOG and F7–F8 electrode pairs, respectively. EOG artifacts were removed using an independent component analysis algorithm (Makeig et al., 1997; Winkler et al., 2011). The Brainstorm software (Tadel et al., 2011) was used to extract peristimulus data from −500 ms (baseline) to +1,000 ms with respect to stimulus onset. Every trial was baseline-corrected to remove the mean (−500 to 0 ms) from each channel. Trials containing large fluctuations exceeding ±100 μV maximum amplitude or 50 μV/ms maximal voltage gradient were excluded from further analyses.

### 2.4. ERP analysis

Two dominant ERP components were analyzed: P300 and MMN. Depending on the regions of the brain in which the activity was most prominent (i.e., regions of interest), the following corresponding electrodes were chosen for analysis: for P300 (maximum peak 200–400 ms post-stimulus), four centro-parietal electrodes (Cz, CP1, CP2, and Pz); for MMN (minimum peak 100–300 ms post-stimulus), four frontal-central electrodes (Fz, FC1, FC2, and Cz). All time windows were based on their grand averages while taking individual variations into account. Baseline corrections were conducted using the 500–0 ms pre-stimulus interval. The amplitudes and latencies of each peak were compared between healthy and tinnitus groups. To display the ERP components, an offline filter (0.5–30 Hz) was applied to the results.

### 2.5. Time-frequency analysis

We used the Morlet wavelet transform to compute time-frequency responses (Herrmann et al., 2005). For the estimation of total activity (which includes the combined contribution of both phase-locked and non-phase-locked responses to the stimulus), the Morlet wavelet transform was applied to individual trials, and the resulting power of individual trials was averaged to obtain total activity. For the estimation of evoked activity (response phase-locked to the stimulus), the individual trials were first averaged, and the Morlet wavelet transform was applied to the averaged (evoked) trial. Since alpha-band oscillatory activity is the most pronounced rhythm in the human brain during relaxed (mentally inactive) wakefulness, we studied whether alpha activity differed between the tinnitus and healthy groups, which could suggest differences in preparatory mental states between the two groups. Furthermore, we also studied EEG theta oscillations, which have been linked to top-down regulation of memory processes (Sauseng et al., 2008). We did not investigate other frequency bands because they did not exhibit observable differences across the experimental conditions. Since the dominant frequency in each frequency band varies per individual, we determined subject-specific frequencies for each band but confined them to be within 8 to 13 Hz for the alpha band and 4 to 8 Hz for the theta band.

To calculate the pre-stimulus total activity in the alpha band, we averaged signal power in a baseline window −400 to −100 ms prior to stimulus onset. To calculate the evoked theta activity, we measured the maximum theta power in the time window between 0 and 500 ms after stimulus onset. All time windows were selected based on their grand-averages. Baseline correction was performed on the evoked theta activity using the pre-stimulus interval −400 to −100 ms prior to stimulus onset. No baseline correction was applied to the total alpha activity since we were interested in studying effects related to the pre-stimulus (i.e., baseline) total alpha activity (Min and Herrmann, 2007). Based on the areas of the brain where the EEG oscillatory activity was most pronounced, three parietal electrodes (Pz, P3, and P4) were selected for spectral analysis. The averaged amplitudes across the selected electrodes were analyzed at their dominant peaks within the corresponding time window (Heinrich et al., 2014; Karamacoska et al., 2019).

### 2.6. Decoding analysis

Deep learning has been tremendously successful, in large part because it enables the automatic learning of discriminative features from the data (LeCun et al., 1989, 2015; Boureau et al., 2010; Glorot et al., 2011). Recently, there has been a growing interest in adapting convolutional neural networks (CNNs) for EEG signal processing (Schirrmeister et al., 2017; Acharya et al., 2018; Lotte et al., 2018; Roy et al., 2019). Deep-learning approaches typically need large amounts of data due to the vast number of parameters that have to be learned (LeCun et al., 2015). Therefore, CNNs do not at first appear to be suitable for a relatively small number of EEG trials. However, a compact CNN called EEGNet was recently been proposed that performs well with relatively small numbers of EEG data (Lawhern et al., 2018). EEGNet is optimized for a small number of learnable parameters and thus reduces the need for additional techniques to deal with limited data, such as data augmentation (Dinarès-Ferran et al., 2018; Haradal et al., 2018; Ramponi et al., 2018; Zhang et al., 2019; Freer and Yang, 2020). That is, it performs well without the need for data augmentation, making the model simpler to implement (Lawhern et al., 2018). In addition, it has been shown that neurophysiologically interpretable features, instead of artifacts and noise, can be extracted from the EEGNet model (Lawhern et al., 2018). For these reasons, we selected the EEGNet architecture over other deep-learning models. Although the basic EEGNet may not result in the best performance (Zancanaro et al., 2021), we opted to use this model because it is a well-established architecture suitable for general applications and interpretations would not be confounded by any complex modifications (Borra et al., 2021; Zhu et al., 2021).

For training and evaluation of the EEGNet model to detect tinnitus patients, we used the time series of EEG data (30 electrodes) of both healthy and tinnitus groups (Lawhern et al., 2018). The architecture and parameters of the EEGNet are listed in both Supplementary Figure 1 and Supplementary Table 1. To investigate which frequency band of the EEG signals contributed dominantly to tinnitus-patient identification, the EEGNet model was separately trained with EEG signals from each frequency band but also using broadband data. The EEG data were band-pass filtered in the following frequency bands: delta (0.5–4 Hz), theta (4–8 Hz), alpha (8–13 Hz), beta (13–30 Hz), gamma (30–50 Hz), and broadband (0.5–50 Hz). In each stimulus-type and task-type condition (i.e., four decoding conditions: target stimuli in the active oddball task, standard stimuli in the active oddball task, target stimuli in the passive oddball task, and standard stimuli in the passive oddball task), the filtered single-trial EEG inputs were used for training and evaluation of the model. To prevent biases in classification performance, the same numbers of EEG data were randomly sampled for both the healthy and tinnitus groups. In addition, to compare EEG decoding performance between the different types of auditory stimuli, the number of EEG trials of the standard stimuli was set to be equal to that of the target stimuli. To compare the decoding performance between pre-stimulus and post-stimulus time windows, the EEG data segmented from 500 ms pre-stimulus to 1,000 ms post-stimulus were additionally decoded in separate time windows relative to stimulus onset: pre-stimulus (500 ms pre-stimulus to stimulus onset) and post-stimulus (stimulus onset to 1,000 ms post-stimulus) periods.

We trained the decoding model for up to 100 epochs, and the best model was finally selected based on the epoch with the minimum validation loss. To evaluate performance, the average of the area under the curve (AUC) in the receiver operating characteristic (ROC) curves, sensitivity, specificity, and accuracy were obtained through the 11-fold leave-one-pair-out cross-validation (Kohavi, 1995; Zou et al., 2007; Pedregosa et al., 2011), in which a pair indicated one healthy individual and one patient with tinnitus. Nine out of 11 folds were used as a training set to train the model, one fold out of the remaining two folds was used for validation, and the remaining one fold was finally used for the model evaluation. This process was repeated 11 times to obtain a total of 11 AUCs of model performance, and the model performance was evaluated through the average of these AUCs. Supplementary Tables 2–4 detail the classification power of the model (sensitivity, specificity, accuracy, and AUC) for each frequency band during the pre-stimulus, post-stimulus, and entire trial period, respectively. In addition, to investigate the cases of misclassifications made by the deep-learning-based model, the misclassified trials from a sample pair of healthy/patient subjects were separately analyzed and compared with the correctly classified trials in the case of EEG alpha activity for the target stimuli in the active oddball task. After the 11-fold cross-validation, the averaged time-frequency representations were computed across all the EEG trials collected individually for the following classifications: true positive (i.e., correctly classified tinnitus patients as tinnitus patients), false positive (i.e., incorrectly classified healthy individuals as tinnitus patients), true negative (i.e., correctly classified healthy individuals as healthy individuals), and false negative (i.e., incorrectly classified tinnitus patients as healthy individuals).

To compare the EEGNet-based decoding performance against other classical machine-learning techniques, a support vector machine (SVM) (Scholkopf et al., 1997; Manyakov et al., 2011) classifier was applied to the current dataset. For the SVM approach, the alpha-band time series of the entire EEG trial at the electrode Pz was used as an input feature to the classifier since alpha activity is generally predominant around the parietal region (Dockree et al., 2007; Cosmelli et al., 2011; Groppe et al., 2013). To investigate the effect of alpha band in the decoding performance of SVM, the AUC was also computed in the case of removing the alpha band, through a band-stop filter, from the input signals. With this feature representation, we applied a SVM with the radial-basis function as a kernel. The regularization parameter and the kernel parameter were chosen using grid search.

To investigate the contribution of each individual frequency band to model training, beyond assessing the classification performance of the EEGNet trained on band-limited data, we also performed a feature analysis of the convolutional layer filter of the EEGNet trained on broadband data (Deng et al., 2021; Riyad et al., 2021). The convolutional layer kernel of the EEGNet model used in this study worked as a temporal filter, which performs a similar role to a filter bank (Ang et al., 2012). Since the sampling rate of the EEG data used for model training and evaluation was 1,000 Hz and the size of the convolutional layer filter was 500, the time window of the convolutional layer filter was 500 ms. To identify the most influential frequencies in the EEGNet model, the input signals, learned filter weights of the first convolutional layer, and corresponding feature maps were projected to the frequency domain using the Fast Fourier Transform (Deng et al., 2021; Riyad et al., 2021). Normalized spectra were then averaged over the 11 cross-validation folds. More specifically, the feature map was computed by a convolution between the input signal and the learned filter weights of the first convolutional layer of the EEGNet model. The spectral power of the eight filters of the first convolutional layer of the present model in each frequency point was normalized by its maximum power over all the eight filters, and the results were averaged across 11 folds. A similar analysis was conducted to compute the spectra of the feature maps.

Last, to investigate the robustness and stability of the deep neural network model, we additionally assessed decoding performance using a smaller number of filters in the first convolutional layer (five instead of eight filters) of the EEGNet architecture. This result is shown in the Supplementary Material.

### 2.7. Statistical analysis

The independent-sample Mann–Whitney *U* test was performed to compare the measures between the two groups (healthy and tinnitus) and to compare the AUCs between the two decoding methods of EEGNet and SVM. To statistically assess decoding performances, we evaluated whether the AUC was statistically significantly higher than the chance level using Wilcoxon signed-rank tests (*Z* scores). All analysis and statistical processing were performed using MATLAB (ver. R2021a, MathWorks, Natick, MA, USA), Python (Python Software Foundation) or SPSS Statistics (ver. 26, IBM, Armonk, NY, USA).

## 3. Results

### 3.1. P300 and MMN

**FIGURE 2:**
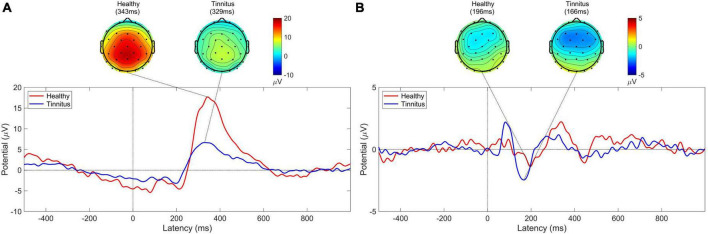
P300 and MMN responses. **(A)** Grand-averaged P300 topographies and ERP time courses at electrode Pz of both healthy controls (red line) and patients with tinnitus (blue line) for the target stimuli during the active oddball task. **(B)** Grand-averaged MMN topographies and ERP time courses at electrode Fz of both healthy controls (red line) and patients with tinnitus (blue line) for the target minus standard stimuli during the passive oddball task.

Significantly higher P300 amplitudes were observed in healthy controls than patients with tinnitus during the active oddball task (healthy group, 18.673μV, tinnitus group, 7.865μV; *U* = 16, *p* < 0.005; Figure 2A). On the other hand, the MMN amplitudes were not significantly different between the two groups during the passive oddball task (healthy group, −3.150μV, tinnitus group, −3.092μV; *U* = 54, *n.s.*; Figure 2B).

### 3.2. Pre-stimulus total alpha and post-stimulus evoked theta activities

**FIGURE 3:**
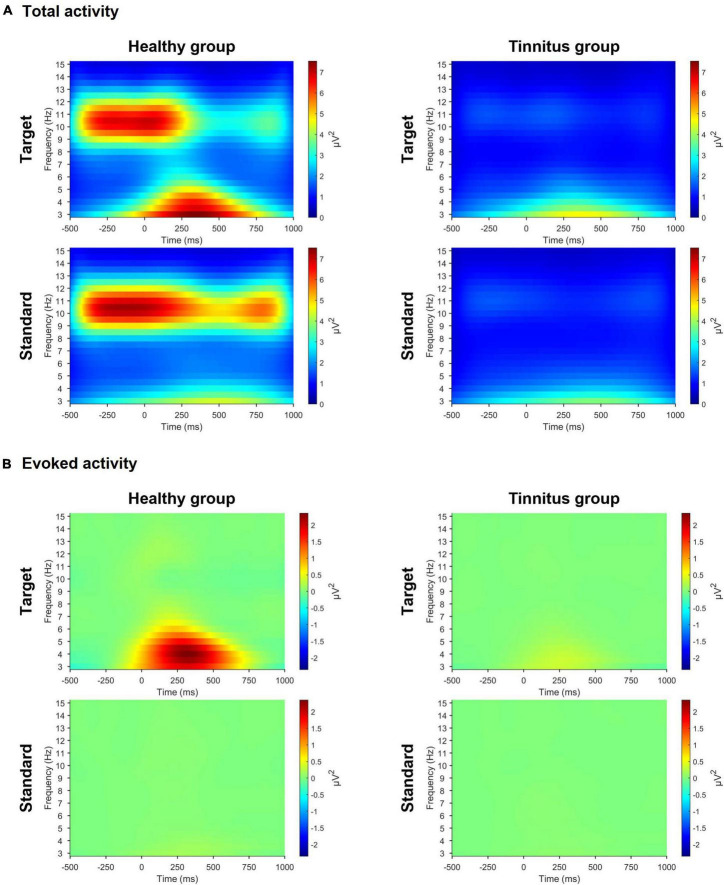
Time-frequency representations in the active oddball task. **(A)** Time-frequency representations of grand-averaged total activity across three parietal electrodes (Pz, P3, and P4) during the active oddball task. **(B)** Time-frequency representations of grand-averaged evoked activity across the same three parietal electrodes during the active oddball task. Note the pronounced pre-stimulus total alpha (8–13 Hz) and evoked theta (4–8 Hz) activities in the healthy group compared with the tinnitus group.

In the active oddball task, we observed significant differences in both pre-stimulus alpha and evoked theta activities between the two groups (Figure 3). For the target stimuli, the healthy group had stronger pre-stimulus alpha activity (healthy group, 4.521μV^2^, tinnitus group, 1.187μV^2^; *U* = 4, *p* < 0.0005) and stronger evoked theta activity (healthy group, 2.431μV^2^, tinnitus group, 0.336μV^2^; *U* = 16, *p* < 0.005) compared with the tinnitus group. For the standard stimuli, the healthy group exhibited salient pre-stimulus alpha activity compared with the tinnitus group (healthy group, 4.747μV^2^, tinnitus group, 1.235μV^2^; *U* = 4, *p* < 0.0005), but the two groups had no significant differences in evoked theta activity.

In the passive oddball task, for the target stimuli, the healthy group had pronounced pre-stimulus alpha activity compared with the tinnitus group (healthy group, 1.702μV^2^, tinnitus group, 0.958μV^2^; *U* = 21, *p* < 0.01), but the two groups had no significant differences in evoked theta activity. For the standard stimuli, the healthy group exhibited salient pre-stimulus alpha activity compared with the tinnitus group (healthy group, 1.693μV^2^, tinnitus group, 0.969μV^2^; *U* = 20, *p* < 0.01), but the two groups had no significant differences in evoked theta activity (Supplementary Figure 2).

### 3.3. Classification performance of the EEGNet model

**FIGURE 4:**
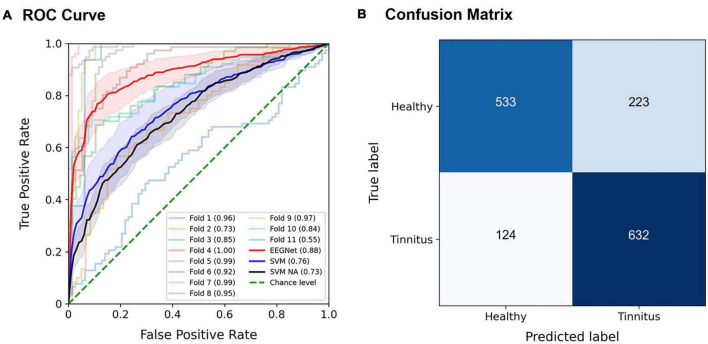
Classification performance of the EEGNet model for the target stimuli in the active oddball task using EEG alpha activity. **(A)** The area under the curve (AUC) scores of each fold in the receiver operating characteristic (ROC) curves were obtained through 11-fold leave-one-pair-out cross-validation using EEG alpha activity in the target stimuli during the active oddball task. Each curve represents the ROC curve for each fold in the cross-validation, with the corresponding AUC scores noted within the legend. The red solid line represents the EEGNet-based averaged AUC across 11 folds, and the blue solid line represents the SVM-based averaged AUC across 11 folds. The black solid line represents the SVM-based averaged AUC based on the removal of the alpha band from the input signals (noted as “SVM NA” in the legend). The green dotted line indicates the chance level. Error bands indicate standard errors of the mean (EEGNet in red, and SVM in blue, and SVM NA in dark gray). **(B)** Confusion matrix of the EEGNet model classification results.

The EEGNet model effectively discriminated patients with tinnitus from healthy controls, most often achieving the best performance using spectral features in the alpha band. For example, when using the entire trial time period, for the target stimuli in the active oddball task, the highest AUC of 0.886 ± 0.042 (mean ± standard error; *Z* = 2.934, *p* < 0.005) was achieved using the EEG alpha band (Figure 4A). The AUCs by the EEGNet indicated marginally better decoding performance than those by the SVM (EEGNet, 0.886, SVM, 0.759; *U* = 34, *p* = 0.08; red and blue lines in Figure 4A). The SVM-based AUC in the case of removing the alpha band from the input signals was 0.73 (black line in Figure 4A). The SVM-based AUC based on the alpha band (0.759) was not significantly different from that of the removal of the alpha band (0.73; *U* = 50, *n.s.*). The associated confusion matrix of the EEGNet model is provided in Figure 4B. Similarly, for the standard stimuli in the active oddball task, the highest AUC of 0.858 ± 0.049 (*Z* = 2.934, *p* < 0.005) was also achieved using the EEG alpha band. On the other hand, for the target stimuli in the passive oddball task, the highest AUC of 0.819 ± 0.068 (*Z* = 2.669, *p* < 0.01) was achieved using the EEG broadband. For the standard stimuli in the passive oddball task, the highest AUC of 0.807 ± 0.058 (*Z* = 2.756, *p* < 0.01) was achieved using the EEG alpha activity.

**FIGURE 5:**
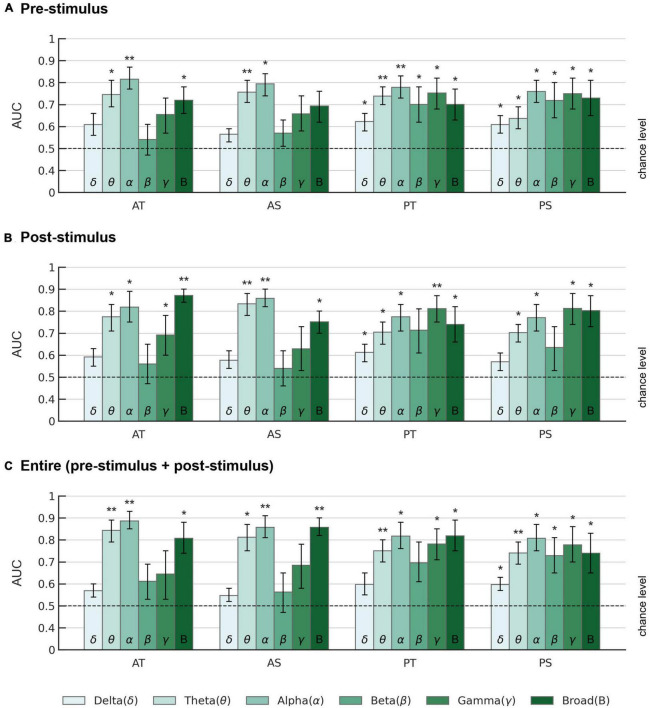
AUC scores of the EEGNet model across different frequency bands. AUCs are displayed for **(A)** pre-stimulus, **(B)** post-stimulus, and **(C)** entire trial period in each frequency band. AT: active oddball task, target stimuli; AS: active oddball task, standard stimuli; PT: passive oddball task, target stimuli; PS: passive oddball task, standard stimuli. Error bars represent standard errors of the mean. The dotted lines indicate the chance level. Asterisks indicate statistical significance (*p < 0.05; **p < 0.005).

Further details for the classification performance of the EEGNet model across all frequency bands, different evaluation metrics (sensitivity, specificity, accuracy, AUC), and different time periods (pre-stimulus, post-stimulus, and entire trial) are provided in Figure 5 and Supplementary Tables 2–4.

Overall, for the pre-stimulus period, EEG alpha activity showed the highest average AUC of 0.815 ± 0.048 (*Z* = 2.934, *p* < 0.005) for the target stimulus in the active oddball task (Figure 5A). For the post-stimulus period, EEG broadband activity showed the highest average AUC of 0.871 ± 0.032 (*Z* = 2.934, *p* < 0.005) for the target stimulus in the active oddball task (Figure 5B). For the entire trial period, EEG alpha band yielded the highest AUC of 0.886 ± 0.042 for the target stimulus in the active oddball task (Figure 5C). As shown in Supplementary Figure 3, these results were largely stable even when changing the number of filters in the first convolutional layer of the EEGNet architecture.

### 3.4. Contribution of each EEG frequency band to decoding performance

**FIGURE 6:**
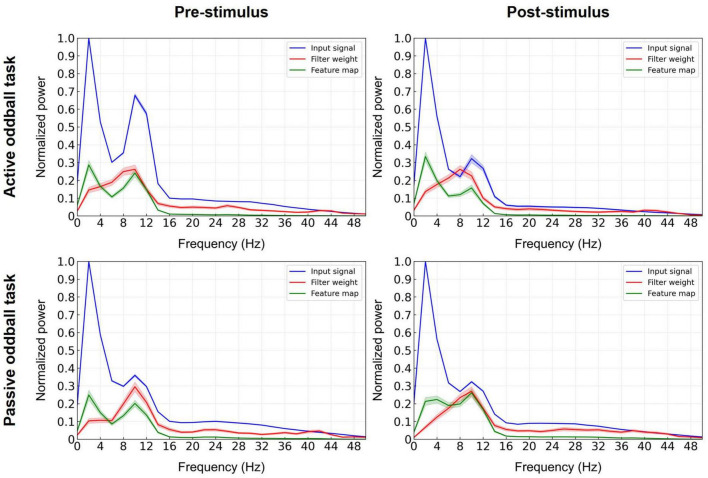
Decisive EEG spectral features in the decoding model. The input signals (blue lines), learned filter weights of the first convolutional layer (red lines), and corresponding feature maps (green lines) of the EEGNet model trained on broadband data were projected to the frequency domain using Fast Fourier Transform (at electrode Pz) and spectra were averaged over the 11 cross-validation folds. Error bands indicate standard errors of the mean.

To identify which frequency band in the EEGNet model with broadband EEG data critically contributed to the performance, we computed the normalized spectral power of the input, the learned filter weights of the first convolutional layer, and its corresponding feature map (Deng et al., 2021; Riyad et al., 2021; Figure 6). The most prominent contribution of the convolutional layer filters was observed in the alpha band. This suggests that the first layer of the EEGNet model enhanced the alpha band at the expense of other frequencies, as also evident by the spectra of the input versus output (feature map) of this layer.

### 3.5. Cases of misclassification

**FIGURE 7:**
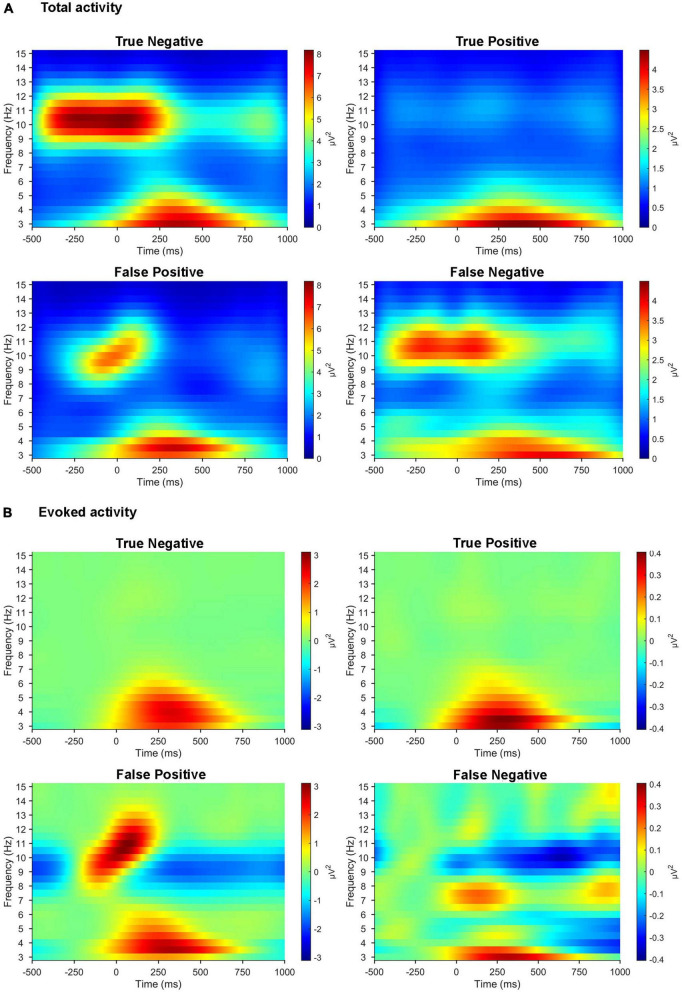
Time-frequency representations of misclassified EEG trials compared with those correctly classified. **(A)** Time-frequency representations of total activity averaged across three parietal electrodes (Pz, P3, and P4) for the target stimuli in the active oddball task. **(B)** Time-frequency representations of evoked activity averaged across the same three parietal electrodes for the target stimuli in the active oddball task. All the plots are computed across all the EEG trials (from a sample pair of healthy/patient subjects) collected individually for true positive, false positive, true negative, and false negative.

We compared the time-frequency representations of the correctly classified versus misclassified EEG trials in a sample pair of healthy/patient subjects (Figure 7). After the 11-fold cross-validation, the averaged time-frequency representations were computed across all trials in the cases of true positive (*N* = 632), false positive (*N* = 223), true negative (*N* = 533), and false negative (*N* = 124). Patterns in the time-frequency plots revealed that the misclassified EEG trials clearly lacked the prominent features of the correctly classified EEG trials.

## 4. Discussion

This study demonstrated that human EEG signals provide promising tinnitus identification features that enable practical tinnitus-diagnostic applications. Based on the EEG spectral analysis, we observed different behaviors of pre-stimulus alpha and evoked theta activities during task performance between healthy controls and patients with tinnitus, suggesting that these spectral components may be crucial features for EEG-based diagnosis of tinnitus. It is noteworthy that the tinnitus group showed significantly reduced pre-stimulus alpha activity compared with healthy participants. Since pre-stimulus alpha activity might reflect the top-down preparation for upcoming stimuli (Min and Herrmann, 2007; Jensen and Mazaheri, 2010), the reduced pre-stimulus alpha power in the tinnitus group may reveal abnormal preparatory top-down processing in the pre-stimulus period. This is consistent with prior studies that found suppressed parietal alpha activity when patients with tinnitus focused on the tinnitus sound rather than when they focused on their own body (Milner et al., 2020). Since the classical P300 and MMN were obviously observed in the active and passive oddball tasks, respectively (Figure 2), the present experimental paradigm seemed to be well designed for investigating each top-down and bottom-up processing. Our results showed more pronounced differences in alpha activity in the active oddball task (i.e., top-down processing) than the passive oddball task (i.e., bottom-up processing). This is probably because suppression of EEG alpha activity is known to be associated with active cognitive processing (Sauseng et al., 2005; Klimesch et al., 2007; Min and Park, 2010), and top-down attention was not focused on the presented auditory stimuli in the passive oddball task (Sarter et al., 2001). Furthermore, since evoked theta activity reflects post-stimulus top-down processing (Sauseng et al., 2008), the absence of evoked theta activity during the active oddball task in the tinnitus group (Figure 3B) also suggests that this group may have compromised top-down processing after stimulation (Hong et al., 2016). It is also notable that the results of time-frequency analyses demonstrated that the misclassified EEG trials clearly lacked the prominent features of correctly classified EEG trials (Figure 7).

In agreement with the EEG spectral analysis, the spectral analysis of the first convolutional layer of the EEGNet model further implicated the EEG alpha band as the most decisive feature for the classification of patients with tinnitus (Figure 6). Further, the significant differences in EEG alpha activity between healthy and tinnitus groups were observed in both the active and passive oddball tasks (see Supplementary Figure 2 for results on the passive oddball task). Overall, our findings consistently point to alterations in alpha band activity as a key discriminative feature in diagnosing tinnitus.

To investigate whether the healthy and tinnitus groups could be distinguished even before stimulus presentation, the EEGNet model was also trained and evaluated by dividing the EEG trial data into corresponding time segments (Figure 5). For the target stimuli in the active oddball task, although the EEG alpha band in the entire trial period yielded the highest AUC of 0.886 (accuracy 0.774, sensitivity 0.827, and specificity 0.721), the EEG alpha activity in the pre-stimulus period also showed a considerably high AUC of 0.815 (accuracy 0.748, sensitivity 0.833, and specificity 0.662). This observation is consistent with the significantly higher pre-stimulus alpha activity in the healthy versus the tinnitus group (Figure 3A). Similarly, during the passive oddball task, pre-stimulus EEG alpha activity resulted in a high AUC of 0.776 (accuracy 0.696, sensitivity 0.750, and specificity 0.642) for the target stimuli.

The SVM-based AUC based on the alpha band (0.759) was not significantly different from that of the removal of the alpha band (0.73) (Figure 4A). This observation suggests that other frequencies also contain relevant information, as confirmed by our EEGNet model in Figure 5, and EEGNet might as well exploit this information to some extent. Although the performance of EEGNet-based decoding was only marginally better than that of the SVM-based decoding (Figure 4A), the use of the deep learning-based EEGNet offers significant advantages over classical approaches such as SVM. This is because deep learning methods are capable of automatically learning complex patterns from data, resulting in improved performance compared to traditional hand-crafted features that require prior knowledge and expertise to select, such as the choice of discriminative EEG channels and frequency bands (Yang et al., 2022). In other words, EEGNet can work directly with raw EEG data, eliminating the need for manual feature extraction (Song et al., 2022).

Symptoms of tinnitus have been linked to hyperactivity and reorganization of the auditory central nervous system (Muhlnickel et al., 1998; Kaltenbach and Afman, 2000; Salvi et al., 2000; Eggermont and Roberts, 2004) with the engagement of other non-auditory brain areas, including the dorsolateral prefrontal cortex (DLPFC; Schlee et al., 2009; Vanneste et al., 2010) and anterior cingulate cortex (ACC; Muhlau et al., 2006; Vanneste et al., 2010). The DLPFC subserves higher-order functions and domain-general executive control functions (Fuster, 1989; Miller and Cummings, 2007; McNamee et al., 2015). The ACC mediates specific functions, such as error detection, attention, and motivation (Johnston et al., 2007; Silton et al., 2010). Both DLPFC and ACC have also been found to be involved in auditory attention (Alain et al., 1998; Lewis et al., 2000; Voisin et al., 2006), thus playing a role in top-down modulation of auditory processing (Mitchell et al., 2005). There is evidence that tinnitus influences affect auditory selective attention (Andersson et al., 2000; Rossiter et al., 2006), with patients reporting concentration difficulties due to their tinnitus (Andersson et al., 1999; Heeren et al., 2014). This is consistent with a study suggesting that a failure in top-down inhibitory processes might play a causal role in tinnitus (Norena et al., 1999). Given that EEG alpha oscillations are linked to top-down processing (Min and Herrmann, 2007; Min and Park, 2010) and inhibitory control of task-irrelevant processing (Klimesch et al., 2007; Min and Park, 2010), our findings provide interpretable neurophysiological correlates of tinnitus that are consistent with prior literature.

Thus, the present deep-learning method of EEG-based tinnitus diagnosis demonstrated its capability of extracting and harnessing interpretable EEG features generally corresponding to known neurophysiological observations. The highest AUC in the alpha band (Figure 5) can be attributed to the difference in alpha activity between the healthy and tinnitus groups observed in the time-frequency analysis (Figure 3). These results were consistently observed, irrespective of the type of experimental task (active or passive oddball tasks). Taken together, these findings indicate that the EEGNet model was trained based on tinnitus-related neurophysiological signatures particularly reflected in EEG alpha activity.

The proposed deep learning-based decoding approach for the identification of tinnitus symptoms could become an effective future technology for the diagnosis or prediction of tinnitus. The training of EEGNet was based on raw EEG data without prior knowledge of important features, which has critical implications when used in practice. However, the present tinnitus-diagnostic approach still has potential for improvement in subsequent studies. A critical limitation is that a larger sample size would have improved the statistical power of our study, but sample sizes were limited by the recruitment of healthy/patient subjects. Thus, despite the use of non-parametric statistical tests (e.g., Mann–Whitney *U* tests and Wilcoxon signed-rank tests), the limited statistical power should be carefully considered when interpreting our findings. Several data-augmentation methods, such as generative adversarial networks (Haradal et al., 2018; Ramponi et al., 2018) or random transformations (e.g., rotation, jittering, scaling, or frequency warping) (Freer and Yang, 2020), may ameliorate the problem of insufficient numbers of EEG data, thus leading to applicable numbers of data for a deep-learning approach.

Overall, our deep-learning approach presents significant advantages over existing methods for tinnitus diagnosis. Our study shows that EEGNet can automatically identify robust task-relevant EEG features, which may facilitate the development of practical and ubiquitous EEG-based applications for disease diagnosis in cutting-edge clinical platforms. Looking ahead, as large amounts of data are progressively collected for heterogeneous symptoms of tinnitus, deep-learning approaches such as the one presented here may prove effective in further discovering stable and generalizable features. Such features may correspond to the varying spectrums of patients with tinnitus, enabling accurate classification and stratification of patients.

## Data availability statement

The raw data supporting the conclusions of this article will be made available by the authors, without undue reservation.

## Ethics statement

The study was conducted in accordance with the ethical guidelines established by the Institutional Review Board of the Hallym University College of Medicine (IRB No. 2016-I013). The patients/participants provided their written informed consent to participate in this study.

## Author contributions

B-KM conceptualized the study and designed the experimental paradigm to identify tinnitus symptoms. E-SH, H-SK, and B-KM performed the experiment and analyzed the data. E-SH, H-SK, SH, DP, and B-KM wrote the main manuscript text and reviewed the manuscript. All authors contributed to the article and approved the submitted version.
