# Supplementary material for: Deep learning-based electroencephalic diagnosis of tinnitus symptom
Source: Front Hum Neurosci. 2023 Apr 26;17:1126938. doi: 10.3389/fnhum.2023.1126938 (PMC10189886; doi:10.3389/fnhum.2023.1126938)
Supplement: Supplementary file 1 [file Data_Sheet_1.docx]

Supplementary Material

Deep learning-based electroencephalic diagnosis of tinnitus symptom

Eul-Seok Hong, Hyun-Seok Kim, Sung Kwang Hong, Dimitrios Pantazis, Byoung-Kyong Min

Correspondence: min_bk@korea.ac.kr

# Deep learning architecture


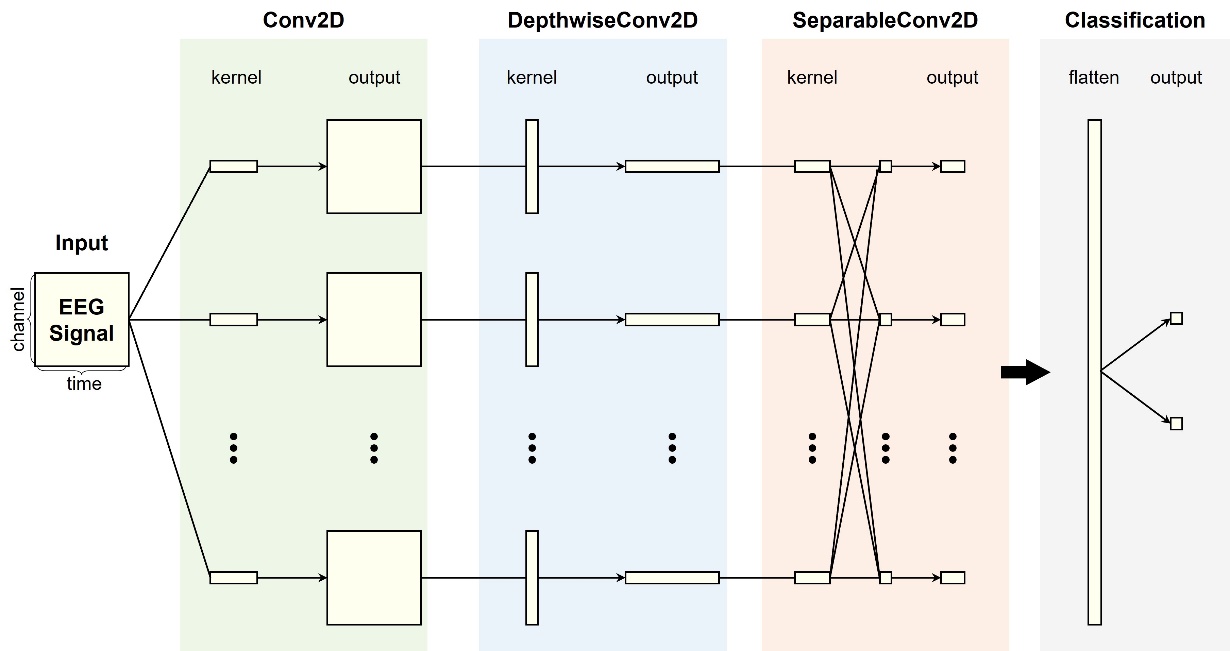


**Supplementary Figure 1.** Visualization of the EEGNet architecture.

The EEGNet model used in this study consisted of three convolution layers as follows: a standard 2D convolution layer, a depth-wise convolution layer and a separable convolution layer. The first convolution layer acts as a band-pass filter, the depth-wise convolution layer as a frequency-specific spatial filter, and the separable convolution layer as an aggregator of temporal features. Last, a softmax layer was used for classification.

The EEGNet architecture parameters were set as follows: C = 30 (number of channels); T = 500 (number of time points for the pre-stimulus period), T = 1000 (number of time points for the post-stimulus period), or T = 1500 (number of time points for the entire epoch period); F1 = 8 (number of filters in the Conv2D layer); D = 2 (number of spatial filters in DepthwiseConv2D); F2 = 16 (number of filters in the SeparableConv2D); N = 2 (number of classes).

To train the EEGNet model, we used He initialization to initialize the model’s weights and Adam optimizer (α = 0.001, decay parameters β1 = 0.9 and β2 = 0.999) to minimize the binary cross-entropy loss function. The batch size and training iterations (epochs) were set to 10 and 100, respectively.

**Supplementary Table 1. Architecture of the EEGNet model.** The Conv2D layer acts as a temporal filter, the DepthwiseConv2D layer acts as a spatial filter for each frequency band, and the SeparableConv2D layer summarizes each output individually and optimally merges them (C = number of channels, T = number of time points, F1 = number of temporal filters, F2 = number of point-wise filters, D = number of spatial filters per temporal filter, N = number of classes).

| **Layer** | **#filters** | **Kernel Size** | **Output** | **Activation** | **Options** | **#params** |
| --- | --- | --- | --- | --- | --- | --- |
| InputLayer |  |  | (C, T, 1) |  |  |  |
| Conv2D | F1 | (1, 500) | (C, T, F1) | Linear | Mode  =same | 500*F1 |
| BatchNormalization |  |  | (C, T, F1) |  |  | 32 |
| DepthwiseConv2D | D * F1 | (C, 1) | (1, T, D*F1) | Linear | Mode  =valid, depth  =D,  max norm=1 | C*D*F1 |
| BatchNormalization |  |  | (1, T, D*F1) |  |  | 64 |
| Activation |  |  | (1, T, D*F1) | ELU |  |  |
| AveragePool2D |  | (1, 4) | (1, T/4, D*F1) |  |  |  |
| Dropout |  |  | (1, T/4, D*F1) |  | P=0.5 |  |
| SeparableConv2D | F2 | (1, 16) | (1, T/4, F2) | Linear | Mode  =same | 16*D*F1+F2*(D*F1) |
| BatchNormalization |  |  | (1, T/4, F2) |  |  | 64 |
| Activation |  |  | (1, T/4, F2) | ELU |  |  |
| AveragePool2D |  | (1, 8) | (1, T/32, F2) |  |  |  |
| Dropout |  |  | (1, T/32, F2) |  | p=0.5 |  |
| Flatten |  |  | (T/32*F2) |  |  |  |
| Dense |  | N*(F2*T/32) | N | Softmax | max norm  =0.25 |  |

# Pre-stimulus alpha and evoked theta activities in the passive listening task


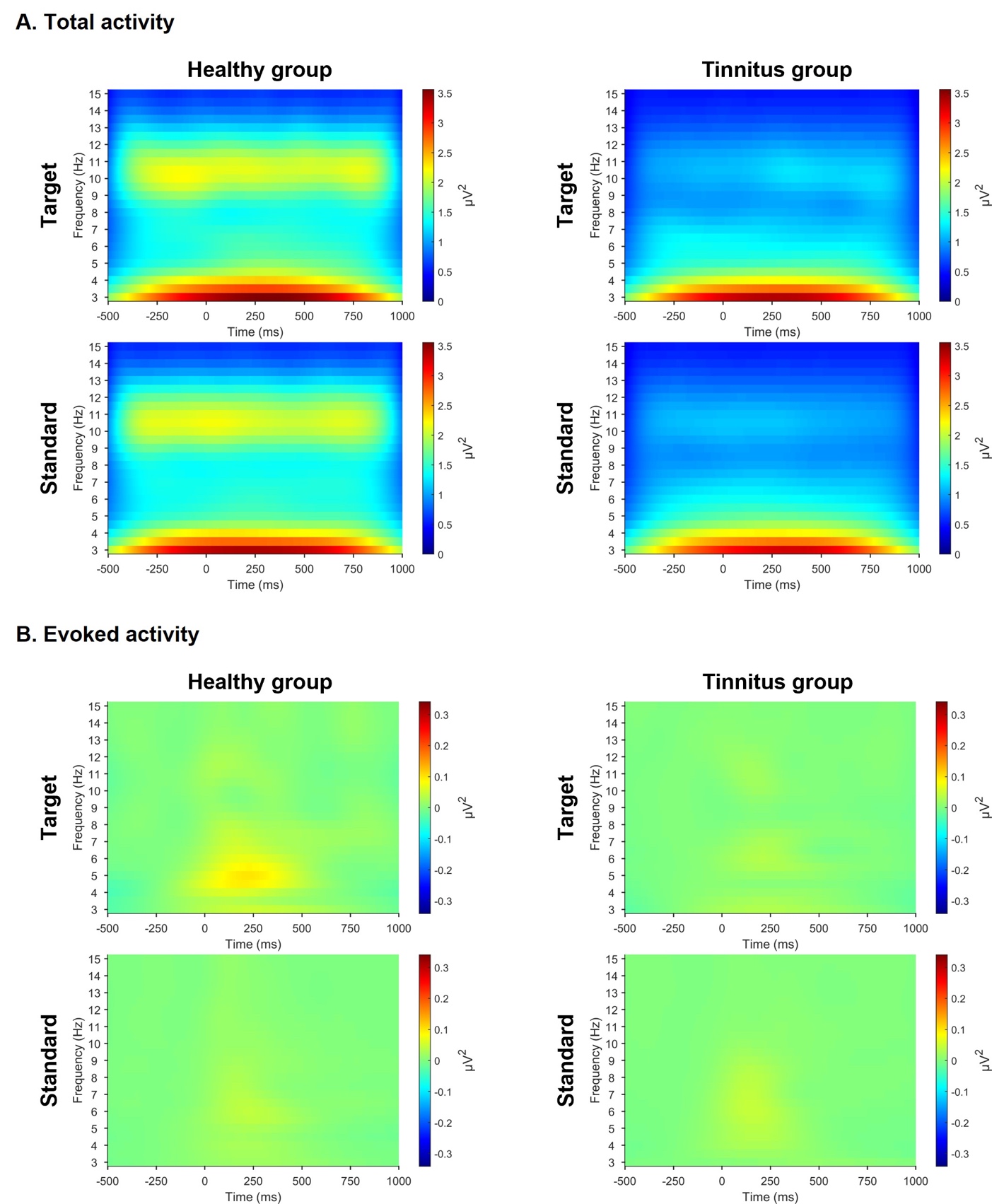


**Supplementary Figure 2.** **Time-frequency representations in the passive listening task** (A) Time-frequency representations of grand-averaged total activity across three parietal electrodes (Pz, P3, and P4) during the passive listening task. (B) Time-frequency representations of grand-averaged evoked theta (4–8 Hz) activity across the same three parietal electrodes during the passive listening task. Note the pronounced pre-stimulus total alpha (8–13 Hz) activity in the healthy group compared with the tinnitus group.

# Classification performance of the decoding model

In signal detection theory, the sensitivity and the specificity are computed as shown below.

|  | | Prediction | |
| --- | --- | --- | --- |
|  |  | Positive | Negative |
| Truth | Positive | True positive (TP) | False negative (FN) |
|  | Negative | False positive (FP) | True negative (TN) |

Sensitivity = TP / (TP + FN)

Specificity = TN / (TN + FP)

Accuracy = (TP + TN) / (TP + FN + FP + TN)

**Supplementary Table 2. Classification performance during the pre-stimulus period.**

| **Condition** | **Frequency band** | **Sensitivity** | **Specificity** | **Accuracy** | **AUC** |
| --- | --- | --- | --- | --- | --- |
| Target stimulus in oddball task | Delta | 0.6693 | 0.4666 | 0.5680 | 0.6096 |
|  | Theta | 0.6159 | 0.7072 | 0.6616 | 0.7448 |
|  | Alpha | 0.8329 | 0.6624 | 0.7476 | 0.8152 |
|  | Beta | 0.4534 | 0.5999 | 0.5267 | 0.5414 |
|  | Gamma | 0.4703 | 0.7256 | 0.5979 | 0.6556 |
|  | Broad | 0.6410 | 0.6706 | 0.6558 | 0.7195 |
| Standard stimulus in oddball task | Delta | 0.6524 | 0.4632 | 0.5578 | 0.5650 |
|  | Theta | 0.6503 | 0.7134 | 0.6818 | 0.7575 |
|  | Alpha | 0.7914 | 0.6385 | 0.7150 | 0.7954 |
|  | Beta | 0.4939 | 0.6052 | 0.5495 | 0.5695 |
|  | Gamma | 0.4031 | 0.8172 | 0.6102 | 0.6589 |
|  | Broad | 0.7094 | 0.5833 | 0.6464 | 0.6936 |
| Target stimulus in passive task | Delta | 0.6830 | 0.5043 | 0.5936 | 0.6218 |
|  | Theta | 0.5983 | 0.7446 | 0.6714 | 0.7372 |
|  | Alpha | 0.7500 | 0.6420 | 0.6960 | 0.7760 |
|  | Beta | 0.7330 | 0.6022 | 0.6676 | 0.6999 |
|  | Gamma | 0.7291 | 0.6403 | 0.6847 | 0.7516 |
|  | Broad | 0.5876 | 0.6915 | 0.6395 | 0.6999 |
| Standard stimulus in passive task | Delta | 0.7772 | 0.3830 | 0.5801 | 0.6087 |
|  | Theta | 0.5314 | 0.6339 | 0.5826 | 0.6361 |
|  | Alpha | 0.7525 | 0.6626 | 0.7075 | 0.7587 |
|  | Beta | 0.7704 | 0.6094 | 0.6899 | 0.7172 |
|  | Gamma | 0.7946 | 0.6061 | 0.7003 | 0.7480 |
|  | Broad | 0.6614 | 0.6879 | 0.6747 | 0.7294 |

**Supplementary Table 3. Classification performance during the post-stimulus period.**

| **Condition** | **Frequency band** | **Sensitivity** | **Specificity** | **Accuracy** | **AUC** |
| --- | --- | --- | --- | --- | --- |
| Target stimulus in oddball task | Delta | 0.6741 | 0.4606 | 0.5673 | 0.5903 |
|  | Theta | 0.7038 | 0.7222 | 0.7130 | 0.7752 |
|  | Alpha | 0.6976 | 0.7818 | 0.7397 | 0.8176 |
|  | Beta | 0.4407 | 0.6685 | 0.5546 | 0.5587 |
|  | Gamma | 0.5089 | 0.7075 | 0.6082 | 0.6938 |
|  | Broad | 0.7716 | 0.7539 | 0.7627 | 0.8711 |
| Standard stimulus in oddball task | Delta | 0.6535 | 0.4441 | 0.5488 | 0.5778 |
|  | Theta | 0.7216 | 0.7459 | 0.7337 | 0.8332 |
|  | Alpha | 0.8272 | 0.7357 | 0.7814 | 0.8593 |
|  | Beta | 0.4516 | 0.6336 | 0.5426 | 0.5387 |
|  | Gamma | 0.3948 | 0.8114 | 0.6031 | 0.6291 |
|  | Broad | 0.5245 | 0.7704 | 0.6475 | 0.7521 |
| Target stimulus in passive task | Delta | 0.7470 | 0.4329 | 0.5899 | 0.6126 |
|  | Theta | 0.5466 | 0.6879 | 0.6173 | 0.7035 |
|  | Alpha | 0.7209 | 0.7188 | 0.7199 | 0.7746 |
|  | Beta | 0.7115 | 0.6199 | 0.6657 | 0.7144 |
|  | Gamma | 0.8149 | 0.6347 | 0.7248 | 0.8115 |
|  | Broad | 0.6016 | 0.7885 | 0.6951 | 0.7380 |
| Standard stimulus in passive task | Delta | 0.7586 | 0.3686 | 0.5636 | 0.5714 |
|  | Theta | 0.5172 | 0.7169 | 0.6170 | 0.7026 |
|  | Alpha | 0.7197 | 0.6706 | 0.6951 | 0.7699 |
|  | Beta | 0.6773 | 0.6183 | 0.6478 | 0.6340 |
|  | Gamma | 0.7997 | 0.7506 | 0.7752 | 0.8111 |
|  | Broad | 0.6961 | 0.7625 | 0.7293 | 0.8041 |

**Supplementary Table 4. Classification performance during the entire epoch-time window.**

| **Condition** | **Frequency band** | **Sensitivity** | **Specificity** | **Accuracy** | **AUC** |
| --- | --- | --- | --- | --- | --- |
| Target stimulus in oddball task | Delta | 0.6106 | 0.4780 | 0.5443 | 0.5669 |
|  | Theta | 0.6504 | 0.8141 | 0.7323 | 0.8442 |
|  | Alpha | 0.8272 | 0.7212 | 0.7742 | 0.8861 |
|  | Beta | 0.4449 | 0.7122 | 0.5785 | 0.6120 |
|  | Gamma | 0.4365 | 0.8050 | 0.6208 | 0.6444 |
|  | Broad | 0.6910 | 0.7910 | 0.7410 | 0.8069 |
| Standard stimulus in oddball task | Delta | 0.6976 | 0.3891 | 0.5433 | 0.5458 |
|  | Theta | 0.6001 | 0.8202 | 0.7101 | 0.8123 |
|  | Alpha | 0.7946 | 0.7242 | 0.7594 | 0.8584 |
|  | Beta | 0.3875 | 0.7566 | 0.5721 | 0.5622 |
|  | Gamma | 0.3920 | 0.8716 | 0.6318 | 0.6855 |
|  | Broad | 0.7321 | 0.7922 | 0.7622 | 0.8568 |
| Target stimulus in passive task | Delta | 0.8354 | 0.3493 | 0.5923 | 0.5974 |
|  | Theta | 0.5707 | 0.7421 | 0.6564 | 0.7501 |
|  | Alpha | 0.6964 | 0.7552 | 0.7258 | 0.8162 |
|  | Beta | 0.7246 | 0.6432 | 0.6839 | 0.6962 |
|  | Gamma | 0.7303 | 0.7614 | 0.7459 | 0.7812 |
|  | Broad | 0.6665 | 0.8319 | 0.7492 | 0.8189 |
| Standard stimulus in passive task | Delta | 0.7841 | 0.3634 | 0.5738 | 0.5980 |
|  | Theta | 0.5473 | 0.7421 | 0.6447 | 0.7414 |
|  | Alpha | 0.7238 | 0.7331 | 0.7285 | 0.8065 |
|  | Beta | 0.8032 | 0.5818 | 0.6925 | 0.7298 |
|  | Gamma | 0.7536 | 0.7497 | 0.7516 | 0.7767 |
|  | Broad | 0.7114 | 0.7162 | 0.7138 | 0.7385 |

**
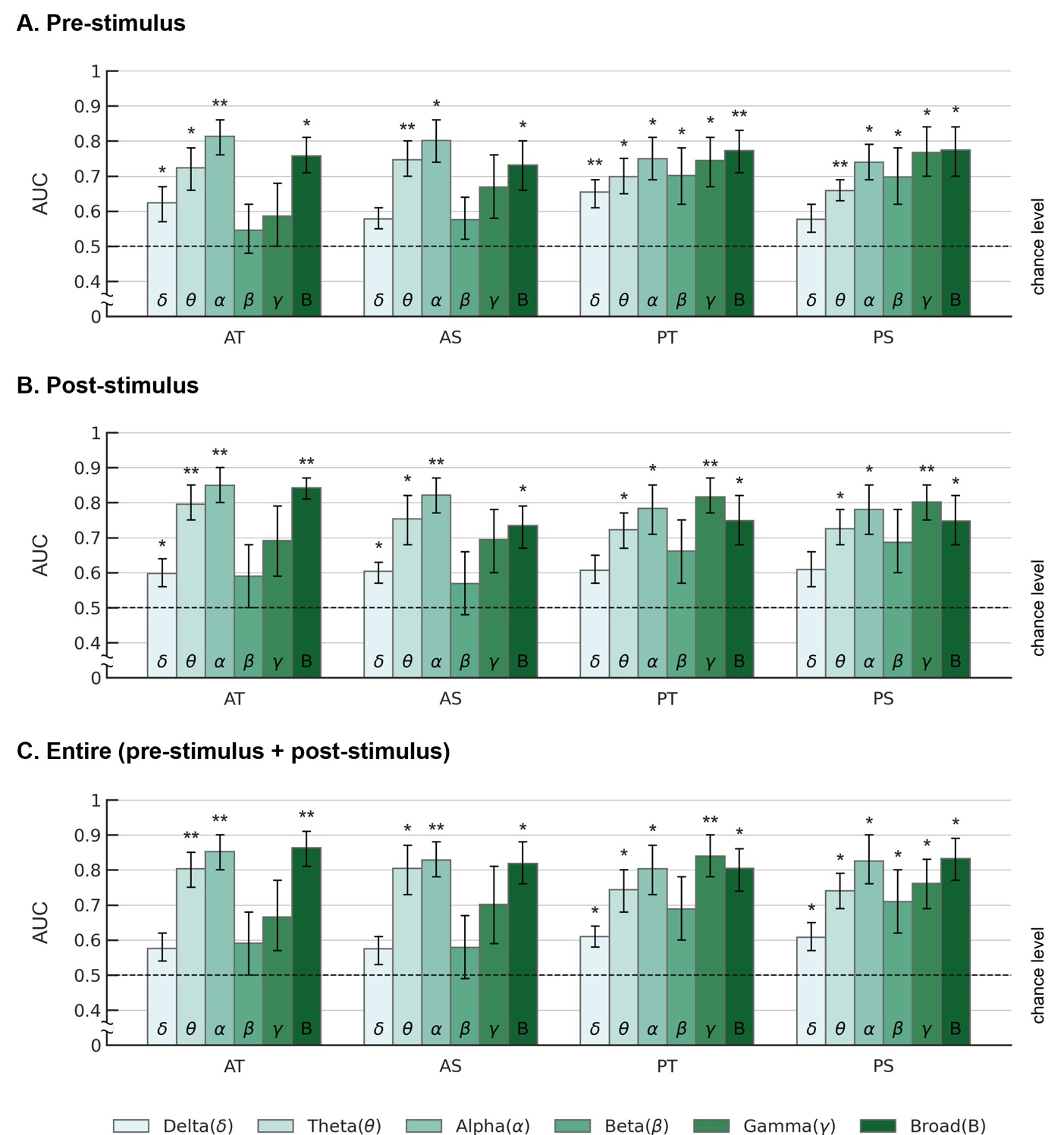
**

**Supplementary Figure 3. AUC scores of the EEGNet model (using only five instead of eight filters in the first convolutional layer) across the different frequency bands.** AUCs are displayed for (A) pre-stimulus, (B) post-stimulus, and (C) entire trial period in each frequency band. AT: active oddball task, target stimuli; AS: active oddball task, standard stimuli; PT: passive oddball task, target stimuli; PS: passive oddball task, standard stimuli. Error bars represent standard errors of the mean. The dotted lines indicate the chance level. Asterisks indicate statistical significance (* *p* < 0.05; ** *p* < 0.005).
